# Improving access to diabetes technologies in children and young people with type 1 diabetes: Healthcare professionals' perspectives and views

**DOI:** 10.1111/dme.70058

**Published:** 2025-04-25

**Authors:** Rachel Dlugatch, David Rankin, Mark Evans, Nick Oliver, Sze May Ng, Julia Lawton

**Affiliations:** ^1^ Usher Institute, Medical School University of Edinburgh Edinburgh UK; ^2^ Institute of Metabolic Science and Department of Medicine University of Cambridge Cambridge UK; ^3^ Department of Metabolism, Digestion and Reproduction Imperial College London UK; ^4^ Faculty of Health, Social Care and Medicine Edge Hill University Ormskirk UK; ^5^ Paediatric Department Mersey and West Lancashire Teaching Hospitals NHS Trust Ormskirk UK

**Keywords:** children and young people, healthcare professionals, inequality, qualitative research, technology, type 1 diabetes

## Abstract

**Aims:**

To understand and explore the strategies, resources, and interventions healthcare professionals are implementing, or recommend implementing, to promote more equitable access to diabetes technology amongst children and young people (CYP) with type 1 diabetes in the UK.

**Methods:**

Interviews were conducted with (*n* = 29) healthcare professionals working in paediatric diabetes in England from (*n* = 15) purposively selected sites. Data were analysed thematically.

**Results:**

Healthcare professionals reported many strategies to help address diabetes technology access disparities in CYP, structured under the following themes: ‘Re‐evaluating staff levels, roles, and efficiency’; ‘Improving communication’; ‘Promoting peer support and community outreach’; ‘Providing financial and social support for deprived CYP/caregivers’; ‘Encouraging CYP/caregiver choice;’ and ‘Funding, sustainability, and burnout.’ Many of these strategies appeared to be local (e.g., site‐specific) solutions, made possible by short‐term, one‐off funding schemes and innovation by individual team members. While some proposed strategies appeared to improve staff time‐efficiencies allowing greater numbers of CYPs to be moved onto technology, others, as interviewees noted, could add to individual team members' workloads and stress.

**Conclusions:**

Healthcare professionals appeared highly committed to addressing technology access disparities in CYP. While some of their recommendations may be easier to implement than others, our findings underscore the importance of adopting a joined‐up, integrated approach to promoting equitable technology access across the UK. This would require closer collaboration and resource‐sharing within and across sites, backed by sustainable, long‐term funding, with a significant portion dedicated to increasing staffing capacity to support the practical implementation of these strategies.

**What's new?**

**What is already known?**

- Equitable access to diabetes technology is crucial for addressing health disparities.
- Inequities in access amongst children and young people (CYP) in the UK are widening along socioeconomic and ethnic lines.

**What this study has found?**

- Healthcare professionals suggested strategies to address inequities, including re‐evaluating staffing, improving communication, addressing unconscious bias, promoting peer support, providing financial/social support, and encouraging CYP/caregiver choice.
- Some strategies may not be sustainable without additional resources.

**What are the implications of this study?**

- Future interventions should focus on sustainability, supported by closer collaboration and increased staffing, backed by long‐term funding.

## 1 INTRODUCTION

Addressing inequities in access to diabetes technology is critical for tackling health disparities. Amongst children and young people (CYP) with type 1 diabetes, lower technology use is associated with poorer health outcomes and suboptimal glycaemic management.^1^ Despite technology uptake increasing amongst all CYP with type 1 diabetes in the United Kingdom (UK), 2020/21 and 2022/23 National Paediatric Diabetes Audit (NPDA) data^2^, ^3^ which are collected annually has highlighted ongoing and widening gaps in technology use between those from the most and least deprived areas and those from white and ethnic minority groups. As a consequence, initiatives have been put in place to help address disparities in technology uptake, including recommendations to offer real‐time continuous glucose monitoring systems (CGM) to all CYP from December 2022, and a National Institute of Health and Care Excellence (NICE) Technology Appraisal (TA943) mandate to make hybrid closed loop (HCL) systems freely available to all CYP in England from December 2023.^4^ Additionally, to help support HCL rollout, one‐off grants were made available to sites where NPDA data revealed particularly large disparities in technology access via NHS England's Diabetes Treatment Technology Fund. While these kinds of initiatives are welcomed, their success is yet to be determined and, as some studies suggest,^5^, ^6^, ^7^ this success will be at least partly contingent on the buy‐in, commitment and availability of healthcare professionals who support technology use in CYP.

In 2023/24 we undertook an interview study with healthcare professionals to better understand, and address, underlying reasons for inequities in technology access in CYP with type 1 diabetes in the UK.^8^ As reported previously, these HCPs offered multi‐factorial explanations for technology access disparities. Alongside inconsistent and inequitable technology commissioning processes, key reasons included: cultural, language, and financial barriers amongst CYP/caregivers (e.g., lack of money to pay for public transport/taxis for CYP to attend clinic appointments); staffing shortfalls, staff burnout, and difficulties keeping up‐to‐date with training to support technology use; and, (un)conscious bias resulting in white and/or CYP from wealthier/more educated families being given opportunities to use technology in preference to other CYP.^8^ In this paper, we seek to advance these earlier findings by reporting the strategies, resources, and interventions healthcare professional interviewees reported implementing, or recommend implementing, to promote more equitable access to diabetes technology amongst CYP with type 1 diabetes in the UK. We also consider the implications of the findings for generating sustainable solutions for supporting equitable use of diabetes technology in this particular patient population.

## 2 METHODS

### 2.1 Recruitment and sampling

Our methods have been reported previously.^8^ In brief, our study design was broadly influenced by Normalisation Process Theory,^9^ an approach that recognises that HCP decision‐making may be influenced and informed by both individual (e.g. a HCP's clinical experience and training) and contextual factors (e.g. availability of funding, the catchment area served by the clinic). We undertook in‐depth interviews with healthcare professionals (consultants/doctors, diabetes specialist nurses, and dieticians) working in paediatric diabetes, whom we recruited from 15 purposively selected sites in England, using an opt‐in approach. We targeted clinical sites identified from 2020/2021 NPDA data^2^ as having particularly high/low uptake of diabetes technologies amongst disadvantaged groups; we also selected sites from across England to ensure geographical variability, and we explicitly targeted those which served above‐average numbers of CYP from low‐income and/or minority ethnic groups. Recruitment continued until there was good representation of different grades of staff from a diversity of sites and data saturation had been attained.

### 2.2 Data collection

Two experienced non‐clinical qualitative researchers (RD and DR) conducted the interviews. These were informed by a topic guide that enabled interviewees to raise issues they considered salient while ensuring discussions remained oriented to addressing our study aims.^10^ Topic guide development was informed by reviews of relevant literatures^5^, ^7^, ^11^, ^12^ inputs from clinical co‐investigators, and revised in light of emerging findings, in line with an inductive approach. Key topic areas explored relevant to the reporting in this article are described in Box 1. Interviews were conducted by telephone or MS teams between October 2023 and April 2024, lasted 1–2 h, and were digitally recorded and transcribed in full.

BOX 1 **Main topics explored in interviews (relevant to the analysis)**

- Interviewee's clinical background, current role, and experience working in paediatric diabetes.
- Characteristics of interviewee's diabetes centre, including: number and types of staff, staff: patient ratios, number of CYP with type 1 diabetes being supported; proportion of CYP from socio‐economically deprived and/or ethnic minority backgrounds.
- Centre's experience of supporting CYP using CGM, pumps and HCL (types of technology interviewee's site is able to offer and support, and why?); proportion of CYP from socio‐economically deprived and/or ethnic minority backgrounds already using pumps/HCL.
- Access to and application of additional funding packages, e.g., NHS England's Diabetes Treatment Technology Fund, to support (and enhance) technology uptake among CYP; examples of how/why interviewee's site has used or would like to use such funding (if applicable); views about use of time‐limited funding.
- Strategies used to introduce and onboard CYP onto diabetes technologies and whether, how and why these have changed over recent years; views about whether and how these strategies have or will change in response to NICE TA943 and 5‐year implementation strategy for HCL.
- Experiences of implementing novel strategies and initiatives to support technology use in underserved populations; how were these devised and by whom.
- Views about whether and how such strategies have helped/hindered technology uptake; lessons learned; other suggestions for strategies which could be deployed in the future to promote equitable technology use.

*Additional topics introduced to explore emergent themes/findings*

- Views about how specific barriers identified by interviewees could or have been addressed by interviewees and their colleagues (e.g., staffing shortages, communication barriers, CYP's concerns about stigma around visible illness; transport difficulties; cultural and financial barriers).
- Experiences of recognising and challenging unconscious bias; views about the role of team dynamics/communication in addressing bias.

Views about longer‐term sustainability of approaches developed within interviewee's site and how sustainability issues might be addressed.

### 2.3 Data analysis

To promote rigour, three highly experienced non‐clinical qualitative researchers (RD, JL, DR) undertook data analysis. The initial analytical phase involved all transcripts being read through repeatedly (data immersion) and cross‐compared to identify cross‐cutting themes.^13^ To minimise the risk of bias, each researcher undertook their own analyses and prepared an independent analytical report, before meeting to discuss their interpretations and reach consensus on a coding framework which captured key themes. To maximise rigour, these interpretations were also sense‐checked with clinical members of the co‐investigator team. As clinical members confirmed that these interpretations resonated with their own clinical experiences, data were coded using the agreed framework and coded datasets were further analysed to develop more fine‐grained interpretations and identify illustrative quotations. NVivo20 (QSR International, Doncaster, Australia), a qualitative software package, was used to support data coding and retrieval.

Ethics approval was granted from the Edinburgh Medicine Research Ethics Committee, University of Edinburgh (23‐EMREC‐007, 18th April 2023). Informed consent was obtained prior to all interviews.

## 3 RESULTS

Our sample comprised 29 healthcare professionals (14 consultants, 9 nurses, 6 dieticians). See Table 1 for further details. Unique identifiers referencing sites (i.e., 001‐015) and roles (i.e., C = consultant, N = diabetes specialist nurse; D = dietician) are used below to safeguard anonymity.

**TABLE 1:** Characteristics of the sample.

	*N* (%)^a^
Sites (*n* = 15)	
Total number of interviewees	29
Interviewees per site—range (mode)	1–3 (2)
Role	
Diabetes consultants	14 (48.2)
Diabetes specialist nurses	9 (31)
Dieticians	6 (20.7)
Years of diabetes experience	
<5 years	1 (3.4)
5–10 years	9 (31)
11–20 years	14 (48.3)
>20 years	5 (17.2)
Gender	
Female	19 (65.5)
Male	10 (34.5)
Age in years: mean, SD (range)	46.5 ± 7.9 (30–59)
Ethnicity	
Asian or Asian British	11 (37.9)
Black, Black British, Caribbean, or African	2 (6.9)
White (British, Irish, South African, Other)	15 (51.7)
Other ethnic group	1 (3.4)

^a^ Percentages do not equal 100% due to rounding.

Findings are structured under the following six themes: ‘Re‐evaluating staff levels, roles, and efficiency’; ‘Improving communication’; ‘Addressing unconscious bias’; ‘Promoting peer support and community outreach’; ‘Providing financial and social support for deprived CYP/caregivers’; ‘Encouraging CYP/caregiver choice’; and ‘Funding, sustainability, and burnout.’

### 3.1 Re‐evaluating staff levels, roles, and efficiency

Interviewees cited staffing shortfalls as a major limiting factor to supporting more CYP to adopt/use technology, particularly those perceived as requiring more staff time (e.g., due to language barriers or low literacy levels) (Table 2). Interviewees further observed that ‘it's a major debate within all diabetes services at the moment, what should the [staff:patient] ratio be’ (008_N), with some raising concerns that current staff: patient ratio recommendations did not take account of the increased demands placed on their time by having to initiate and support technology use in a wider group of CYP (Table 2). Hence, to support a national rollout and ensure equitable access, interviewees at many sites described wanting and needing to recruit additional clinical staff, especially nurses, dieticians, and psychologists, with some also questioning whether there should be higher staff: patient ratios in sites serving disadvantaged communities, where ‘families [who] definitely do need more support…should get more of our time’ (009_D).

**TABLE 2:** Supplementary quotations.

Themes	Sub‐themes and additional illustrative quotations
Re‐evaluating staff levels, roles, and efficiency	***Staffing shortfalls disadvantaging CYP from disadvantaged backgrounds*** …if you put a white, English‐speaking, middle‐class family who will get it in two hours and it's going to take you eight hours to do it with a family who don't speak the language, who are not interested in doing it because they think it's too much, because they've got literacy skills, you think, *Well, I could do four patients* versus *one patient*. Then that's what you're going to do, isn't it? (010_C) ***Staff: patient ratios*** …we've got 166 [CYP] on our current caseload and that's increasing and there's only…two full time nurses to that. … we've recognised a gap in the service, we're getting left behind to our peers. And we need to think about the bigger picture…it's only going to get more technologically advanced. (003_C) ***Expanding teams to include more non‐clinical staff*** *Diabetes care technicians* …a data manager for example, that would be quite useful as well. Because we do spend a lot of time chasing people to, you know, upload pumps…[So] it's really just to collate the data for us before clinics, because that takes such a lot of time. And then it takes away [clinicians'] time from face to face, or actually looking and interpreting the data. (006_D) *Youth support workers* …we are looking at a business case to have a youth worker to help with young adolescents to improve the technology uptake as well…I think a youth worker is mainly going to be beneficial for the teenage population for having more engagement outside of the clinic environment…[as] there are some units who have said they have been beneficial and they showed evidence that it's improved engagement in the clinic attendance, improvement in the HbA1c outcomes. (008_C) ***Using existing staff time and resources more creatively*** *Virtual approaches to service delivery* …from Covid we started a virtual training programme…to reach over 300 patients in schools, nurseries and colleges…which actually worked really well. So we've got a really good virtual school training pathway. (012_N) …now we've got alternative ways of working. So, you know, spending a home visit, before, you could be there two or three hours. Where, if you did a virtual meeting, you could be done in 20 min, half an hour. (003_N) …the pump initiations we've been doing online as well because it's better attendance on Teams…So, again, for some families that might be a bit of a barrier as well, accessing on Microsoft Teams, so we decide which families might be more suitable for online initiation and which families need face‐to‐face appointments…I think because it's easier to do online, I think we've managed to get more numbers through. (008_C) *Group‐based approaches* …if we know some families are really tech savvy, we wouldn't put them in a pump start with a family that we know struggles with technology…we'd put like‐minded families in those pump starts. (001_N) *Using company reps* We [clinicians] don't all need to be there in a pump start because, at the moment, all three of us are there to support all the patients. So, luckily, with the reps in attendance for these pump, or these technology starts, it gives you the chance and the opportunity to just be there and let the rep do all the delivery, and you can just do all the paperwork in the background doing the contracts, doing the education checklist…rather than have all three members of staff taking up that time. (003_N) *Freeing up time to support CYP/caregivers with complex needs* …when COVID hit we moved the same [CGM] programme online…[It's] not for everybody and we still had to do one‐to‐one, face‐to‐face, for the people who required interpreters, and the people who had educational challenges. But for the 70 or 80 per cent who could do it online, it just saved a massive amount of time…And the ones who need your support, it frees up the time to give them the one‐to‐one support that they need. (007_D) …it's partly us offering pumps to people we know never would have [been] offered them before. So families that aren't carb counting…we'd probably as a team would have said: actually, the pump isn't suitable for them, because they wouldn't pass the pump assessments…From a kind of a dietetic point of view, especially with…families that don't and can't carb count, we're looking at…taking photos of their meals, we're getting average carb values for meals, we're making a bit of a plan for what we can do with when they get on the pump. (015_N)
Improving communication	***Using resources to facilitate communication with people who do not speak English*** We would use Digibete, which is an NHS paid‐for platform for education in young people…[which includes] video's that have been translated in different languages. So we would use that to get essentials across. (014_D) ***DigiBete shortfalls and limitations*** And interestingly we were looking on DigiBete to see what their videos were like for pumps and tech. And again, the Black and Asian community are not very well‐represented. (015_C) ***Challenges securing interpreters*** So if English isn't their first language and they need interpreting services, particularly if the child is younger, that can be really difficult to manage. Some people speak a very specific dialect of a particular country and you can't always get an interpreter so you bumble along. (007_C) The only time we tend to struggle is if it's a sort of more specialist language, like the family that I said that were from Eritrea there, Tirgrinya is quite a niche language, so we have to make sure that's booked well in advance of the appointment, so that they can attend. (010_N) ***Communicating* via *text*** If you phone them, they don't understand what you're saying, but they can…use things like Google Translate to decode messages…They are the skills that they already have, just for living life and surviving. They can use those if you get the information to them in the right format. (007_D) ***Using video‐based resources*** So what we have done is we recorded a number of videos. So the ones we recorded are like a insulin pump demo, we're showing all the options we have, you can see the actual pumps, the pros and cons of the different pumps, as well as bit of an introduction to pump therapy in general. So that's sent to all the families that you know, express interest in progressing towards a pump. (015_D) Try to use as much video‐based technology as possible because pictures obviously explain things a lot simpler than a lot of words do. So within our education, our teaching programme as such, it's a mixture of text and links to videos to explain the concepts. (009_D)
Addressing unconscious bias	***Reviewing NPDA data and recognising bias*** I just said that because our NPDA data was very clear and we went through a quality improvement project as well on this particular issue [of bias]. And, yes, so I spoke about it in the team meeting and said that, you know, we cannot go on like this, we need to address this issue properly. (012_C) We sat down and went, *why is this happening*?… So, I think once we did the data and we were honest that there was a bit of unconscious bias going in, there's a lot of barriers. How can we remove these barriers? (007_D) ***Challenging bias within the team*** …as a team now we challenge each other…because we bring [the team] in the meeting and say, you said that look at all these families there, they will benefit from pump, [but] I do not know whether the mum has got so much mental health issue…So we challenge now…why do we think that [they can't cope]? Why can't we try this? So as a group we now are become more conscious. Previously we would have said, *Oh they can't cope [with a] pump*. (011_C) …it's just about having that team dynamic and that team thinking to try and address any sort of bias that you may or may not have within your team…to just make sure that everyone is coming through with the same sort of reasons for going onto technology following the national criteria and just offering it to everyone who is eligible. (005_C) Over the years we've worked very hard ourselves to not let our biases say that…they won't cope. (009_C) ***Cohesive team‐working*** If they're not on a pump or CGM, we always ask ourselves why and try and revisit whether there's any criteria they can meet, discuss it, and offer them the technology if they're eligible to see if they would want to go onto it. Afterwards, we then discuss it within the post‐clinic MDT meeting and make sure that the team are in agreement that they do meet the criteria before we say we will add them onto a waiting list for either a pump or CGM. (005_C) ***Biases further challenged by seeing CYP do well on technology*** We're seeing, with families and patients that we have that have started [using a pump or HCL], that a few years ago you might have thought, *Well, they wouldn't be able to manage a pump*. [But] they're doing fine on it…So as soon as we see a few successes, I feel like it just changes how we work. (009_D) ***Eliminating unnecessary paperwork and assessments*** So we had a pathway to start children on the pump. So what we do is we discuss the pumps we offer in the clinic and then the families agree and then we put them through a quiz okay…So we already we have bias actually because this quiz could only be done by people who are from upper socioeconomic groups, people who are educated, people who have got good English knowledge, people with good English knowledge. Already there is so much bias actually…So what we did was we completely scrapped off this assessment. Completely scrapped off, okay, assessment and said we don't need to do that actually. Because already we are prejudiced, we are biased actually. (011_C) ***Technology upon diagnosis*** This month we've started CGM on diagnosis for all newly diagnosed children…So even if their parents don't speak English or if they're not educated, they will all get technology on diagnosis. I think that's going to be a real benefit…they don't need to research and then come to ask us. They will get it. (009_D) We get children onto CGM or Libre within either a few days of diagnosis or certainly within a week or two. So we know that we're moving technology onto these children right from the very beginning…So we put children like that onto pump therapy very quickly, within maybe two, three weeks of diagnosis. If it's your motivation and we've got the technology and we're allowed to fund it, it's not necessarily a giant leap to consider should we get these children onto pump therapy and closed loop systems? It gives them freedom of choice. It gives them better quality of life. (008_N) ***Feasibility of onboarding to pumps at diagnosis*** It was a national meeting and there was quite a lot of attendees, and in that, she [clinician at a well‐resourced site] mentioned…she's been doing what she calls pump on diagnosis, and she's giving anybody who has been diagnosed three months to be on hybrid closed loop pump therapy from diagnosis… She's got amazing resources and an amazing team, don't get me wrong. I think we would struggle; we would struggle with that in fairness. (003_N)
Promoting peer support and community outreach	***Peer support for adolescent‐aged CYP*** It's the teenage population that we really struggle to engage with these things…we want to try and get them together and show them that they're not the only one and there's all these other people out there and how they're coping with it. Obviously we can introduce them to other young people using technology and see if that helps. (004_N) …we were thinking about…a[n]…event…where we try to target maybe teenage girls, especially maybe Asian teenage girls that…would maybe come to a day event to get involved and make it fun…when it's fun they learn a lot from being with each other. (009_D) …we tried to group them, so children from the same ethnic minorities come together so they talk to each other more freely. We invited some families from those minority groups as well who are really doing well and much better educated, so they act as a peer support. (008_C) ***Video testimonials*** So we've got video testimonials from various people. As we've gone along, we said, *would you mind doing a short video of what it's meant to you?*…So…if someone's unsure about things, we'll bring them in, we'll show them the systems and we will also say, this is what it's meant to so and so…and then it may just speak to them in a way that allows them to understand it more effectively. (007_D) ***One‐to‐one peer support*** A lot of positive outcomes we've had, has been through peer support…I've got one young girl from Africa. She would not wear a Libre, because it had to be worn on the back of her arm and other people would see it in the changing rooms…And she goes to a school where we've been able to get her to meet another young girl…who's on an insulin pump, and so they've had a couple of meetings…and been able to talk about things, and I've managed to persuade her to…potentially to go onto a pump. (010_N) ***Bridging cultural divides by involving locally respected figures*** …in the past what we have often done is we've done work with the local mosques and the Imams. They can be very influential in promoting healthy living…So you kind of work with whoever you can gain access to. And, if within the Asian community, the mosques and the Imams can be an influence, then sometimes it's worth making contact with those areas. (008_N) ***Involving celebrities to normalise wearing visible devices*** Introduce them to families where…the children sometimes see that it's a normal thing to have a pump or a sensor … if they're a footballer or a rugby player, have you seen so‐and‐so, he wears the [sensor] or the teenage girl…you know, Lila Moss, who's a model, who's got a sensor. (002_C) Encourage that peer support and develop it to the extent that calling in celebrities with type 1 diabetes. And talking to these populations and telling them not to be scared about opening up and taking help from others. (012_C)
Providing financial and social support for deprived CYP/caregivers	***Providing meals for families*** There are people in the trust that run this forum and they've been doing work on having meals in the hospital available for parents of children. Like they have this thing now that they're doing where they have frozen meals available for parents to heat up these meals…So they can have meals when they're in the hospital or they have them at nighttime or something, parents can access things. They also do food bank referrals. (009_D) ***Accessible transportation and reimbursement*** Transport can be a bit of an issue. So you know, a lot of our families wouldn't drive. So they're reliant on buses and trains to get to us. But that's not always the most reliable. So even attendance at appointments isn't always the easiest. (015_D) I also think they're unable to pay taxi money…It just wouldn't make sense to try and get a bus anyway because it might take them 40 min to get from somewhere that's actually not too far away, on two different buses. So they just end up getting taxis…I have a family that the girl has diabetes, the baby brother had cancer so they were needing to come to hospital quite frequently. The dad really struggled with parking and everything and actually coming to appointments. So I did ask if they did have any of these parking vouchers left or if they were still doing it but I haven't gotten anything back so I don't know if they can [cost] that. (009_D)
Encouraging CYP/caregiver choice	***Elective admission***
	We'll bring the child in for a full week, or four to five days, and we'll start the pump as an admission instead of outpatient…it might be a family…if they need a bit more reassurance, a bit more monitoring, a bit more help or a bit more intensive education at the beginning. I think that's been a real change in our service as well in the last few years and it's worked really, really well. (009_D)
Funding, sustainability, and burnout	***Commitment to addressing inequities in access to technology*** …as a team, kind of especially since we saw those last [NPDA] results, we have regularly in our kind of our monthly meetings tried to make a plan as to actually how we could make it more equitable and more fairer. (015_N) ***Staff working beyond contracted hours*** The nurse trying to access charity phones is very dedicated and spends a lot of time beyond her normal hours to obviously help these families. (013_D) I'm meant to be off on Friday, but I work most Fridays. And I'm always late picking my kids up, and end up working when I have picked them up. So that's a knock‐on effect as well… you work in the NHS because you want to make a difference. (012_N) ***Staff burnout*** I'm worried about our service because of that. You know, that's why our consultant went off, because she was knackered. She's burnt out. She's done this for years, and it's just the pressure is continuous. (012_N) ***Staff declining additional weekend work*** We did have the extra money available [from the one‐off NHSE grant] to pay for staff to maybe work weekends to do education sessions or pump starts sort of thing. What we found is the staff had enough time within their work period because it was benefiting them just to do it within their work hours and they didn't want to do the extra work, which I wasn't blaming them for, you know. They need their time off as much as anything to prevent burnout. (007_D)

Interviewees across the sites also suggested that expanding their teams to include non‐clinical staff could help improve technology uptake. Some, for instance, reported hiring or wanting to hire staff such as administrators who could be ‘ordering devices, organising training, organising group sessions’ (002_D) and/or diabetes care technicians to help, for example, with setting‐up/managing technology‐related accounts/passwords and accessing CYPs' data (Table 2). While only a minority reported having access to youth support workers, virtually all described wanting to employ such individuals to help improve attendance and engagement, particularly amongst teenagers, thus increasing the likelihood of a wider group of CYP using technology (Table 2).

While interviewees considered increasing staffing levels to be a priority, many reported having no budget to hire new staff and, hence, having to use existing staff time and resources more creatively to support increased technology uptake. Interviewees, for instance, described making greater use of virtual approaches, including offering remote instead of in‐school education to staff, doing some ‘home visits’ virtually, and delivering some elements of CGM/pump training via self‐completion online modules (Table 2). Additionally, many reported increased use of group‐based approaches to deliver pump education and training to CYP/caregivers (with careful thought given to the composition of groups to accommodate different learning capabilities (Table 2)), and inviting company representatives to help deliver this training to free up staff time/capacity (Table 2). These strategies, as some interviewees further noted, enabled them to focus more time and energy on CYP/caregivers with complex educational and support needs (Table 2), such as those needing help with carbohydrate counting (Table 2).

### 3.2 Improving communication

Interviewees described how their difficulties communicating with certain CYP/caregivers (e.g., those perceived as having low literacy levels and/or who did not speak English) could negatively impact technology uptake. To help address these difficulties, interviewees at some sites serving deprived and/or ethnically diverse populations reported using Digibete, an online digital platform commissioned by NHS England, to offer technology education/training to CYP/caregivers, including those who did not speak/understand English (Table 2), whilst acknowledging some of its shortfalls (Table 2). Where funding was available, some also described arranging translation of educational materials into CYPs' and/or their caregivers' most common languages, which, notably, differed between sites. In doing so, interviewees noted the high cost of professional translation services. Some also noted the challenges of translating materials into all relevant languages used by CYP/caregivers attending their clinics: ‘we're dealing with up to 22 different languages within the area’ (008_N).

While interviewees reported being able to book interpreters for some in‐person and phone appointments, they also highlighted challenges to securing interpreters to accommodate certain languages and specific dialects (Table 2). Additionally, interviewees emphasised the need to communicate outside of appointments to support diabetes technology uptake and use, and some reported implementing creative strategies to make this possible. This included communicating via WhatsApp or other text‐based platforms, which, as interviewees suggested, allowed CYP/caregivers (or other family members with more advanced English language skills) to translate text and slow down the pace of conversation, which could help improve overall communication (Table 2).

Additionally, some observed how it was especially helpful when they or another team member spoke the same language as the CYP/caregiver. Hence, when possible, they described trying to coordinate appointments so that CYP/caregivers could be matched to an appropriate multilingual healthcare professional:

I speak Hindi, Urdu, Punjabi; a lot of the Asian families, if the parents are not comfortable with English, I almost cohort them so I end up seeing more of them. (010_C)

To better cater for those perceived to have limited literacy, interviewees at some sites also noted how they or their colleagues had capitalised on existing skillsets within the team to create pictorial and/or video‐based (Table 2) educational resources:

We refined those initial massive workbooks down to these two A4 pages…we use a lot of visual representation graphics, so smiley faces, graphs, colours, to represent information in a more easily accessible format for anyone. (007_C)

### 3.3 Addressing unconscious bias

Interviewees reported how, as a result of reviewing NPDA data, they had become more cognisant of technology inequalities within their own cohorts (Table 2), as well as their own biases when (not) recommending technology to certain CYP. These interviewees noted benefits to questioning and challenging these biases through group discussions (Table 2), alongside cohesive team‐working. This included convening (weekly) multi‐disciplinary team meetings, reviewing individual CYP regularly to assess technology readiness, and drawing up agreed plans of action to ensure CYP/caregivers received consistent messages about how, and why, they might benefit from using technology (Table 2). Many described having a more liberal approach to recommending technology as a consequence, with some noting how observing CYP succeed on technology had helped challenge their earlier assumptions about who might struggle (Table 2).

Some also reported taking proactive steps to promote equitable access by eliminating unnecessary assessments and paperwork for CYP/caregivers (Table 2). In addition, interviewees at some sites reported shifting towards initiating technology use at diagnosis (Table 2). As these individuals suggested, this inclusive approach helped mitigate risks of better informed/educated parents accessing technology first due to having greater knowledge and confidence lobbying for it (Table 2). Others at smaller, less well‐resourced sites, however, questioned the feasibility of such an approach (Table 2).

### 3.4 Promoting peer support and community outreach

Peer support, according to many interviewees, was one of the most effective ways of improving technology uptake amongst reluctant CYP. Interviewees described targeting this kind of support at adolescents and/or CYP/caregivers from certain ethnic minority groups who expressed concerns about stigma or their peers asking invasive/personal questions if they saw them wearing a pump or CGM device (Table 2). They described how recording and using video testimonials of CYP speaking about their positive experiences with technology (Table 2), in addition to more direct peer support (e.g., connecting CYP one‐to‐one in real time), could be particularly effective (Table 2).

Furthermore, several said that connecting parents via family support groups could also help to improve technology uptake:

The parent will say, *I can sleep, and I haven't slept in five years*. [They're] hearing someone actually that's done that and going through it. To say something like that is much more powerful than us saying…[technology] will improve your [glycaemic] control. (015_D)

Because traveling to clinic was challenging for many CYP/caregivers, and CYP/caregivers often felt more at ease in familiar places, some interviewees reported successfully undertaking ‘technology roadshows’ (012_N) and other outreach work when provided a one‐off grant from NHS England:

We hired a room and we got all the reps from different companies to come. We had some interpreters there. And we had, we identified some of the ethnic minority patients who already had a pump…We had almost 20 families to this event because we spent some time speaking to them in their language. And then through this project…we were able to put 14 children on pump. (011_C)

Others added that involving popular or locally respected figures, such as athletes, celebrities, or Imams, could help promote technology access by bridging cultural divides (Table 2) and/or normalising wearing visible pumps/sensors (Table 2).

### 3.5 Providing financial and social support for deprived CYP/caregivers

Interviewees observed that socioeconomically deprived CYP/caregivers could have competing demands on their time, making it more challenging to prioritise their/their child's diabetes:

And the truth is…if you're impoverished…you're struggling with your basic needs, struggling to get enough money to buy food, to pay the heating…of course you can't concentrate on the basic cares of diabetes…We have concerns about putting children on closed‐loop…or pump. (002_C)

To free up CYP/caregivers' capacity to focus on diabetes‐related care, some reported assisting those facing financial and/or social hardships which, they suggested, ‘restricts them from access to something like technology’ (002_C). This included using a support worker to ‘help them to see what other benefits [they] could be entitled to’ (009_C), facilitating access to free food and/or a food bank (Table 2), or making (potential) use of a social worker to assess social and familial needs (e.g., child support, childcare, parenting education).

Interviewees at some sites also reported providing or attempting to source diabetes technology‐compatible phones or SIM cards for those who could not afford them. In some cases, this involved staff approaching charities to access refurbished phones; another interviewee described attempting to establish an in‐hospital recycling program, wherein old hospital phones were recycled and donated to CYP/caregivers in need:

I've asked the Trust if we could [have] any devices that come back in the Trust that are old…work mobile phones for example. If they go back into the Trust, what happens to them? Could we be the first refusal? (012_N)

Finally, because getting CYP/caregivers to clinic was described as critical to their diabetes care and ability to use technology, interviewees highlighted the need for accessible transportation and/or parking. While some reported that their sites reimbursed bus fares, some further noted that bus routes were inconvenient and prohibitively long, and highlighted the need for provisions to reimburse other travel expenses (e.g., taxi fares) for those on low incomes (Table 2).

### 3.6 Encouraging CYP/caregiver choice

Interviewees acknowledged that CYP/caregivers have their own unique circumstances and preferences and described how expanding patient choice could positively influence technology uptake. For example, interviewees at a few well‐resourced sites highlighted the importance of offering elective, inpatient admissions for technology starts rather than only having the option to start technology on an outpatient basis. These interviewees noted how, with this additional clinical oversight, nervous CYP/caregivers, who might otherwise have declined technology (e.g., due to safety concerns arising from being non‐native English speakers or having low literacy), felt confident enough to transition onto technology (Table 2).

Additionally, some noted how offering a choice of systems (e.g., Medtronic, Omnipod, Tandem Control:IQ), could also help improve uptake. In particular, some emphasized the importance of offering CYP the option to use discreet devices, to help address concerns about stigma, with many noting the popularity of the Omnipod5 due to its wireless design: ‘Now it's very discreet so they don't have to inform the extended family. Or if they go to mosques and temples’ (011_C).

### 3.7 Funding, sustainability, and burnout

While interviewees expressed a strong commitment to addressing inequities in access (Table 2), many, especially those in sites with lower staff: patient ratios, also questioned the long‐term sustainability of some adopted approaches. Some, for instance, noted how their initial success getting CYP from underserved communities onto technology had been made possible by the goodwill of staff working outside their paid hours (Table 2). Others raised concerns about staff burnout/sickness due to ‘the pressure [being] continuous’ after being labelled as a ‘failing service’ (012_N) and/or the (potential) need for staff work in the evenings/weekends to participate in outreach work.

Indeed, some described how team members declined involvement in paid opportunities to participate in outreach work due to worries about exhaustion and burnout (Table 2). Others questioned how to launch community‐based initiatives, like residential weekends and community cafes, without turning to fundraising, while those who had received short‐term grants from NHS England questioned their ability to sustain their efforts after such funding came to an end. This included 012_C whose site had used this funding to appoint a diabetes educator on a one‐year contract and who noted that ‘once that funding is exhausted…it will cause a huge amount of problems’ (012_C) and 003_N who reported how:

The NHS England push…a lot of our units…were offered funding for increasing access…especially in minority ethnic and deprived communities. And we were given this funding to work extra hours to get more patients on pumps…which we did…but I think the biggest challenge is…how we, as specialist nurses, dietitians, consultants [will] handle the actual demand in terms of pressure, in terms of our own mental health and wellbeing, burn‐out. (003_N)

## 4 DISCUSSION

Interviewees proposed a variety of strategies to help address inequities in access to diabetes technology in CYP living in the UK. A key suggestion involved increasing clinical staffing levels, especially at sites serving high numbers of CYP from underserved communities.

Given the significant and growing financial pressures within the NHS which interviewees alluded to in their accounts and which have been described as contributing to a ‘workforce crisis,’^14^ this recommendation, while extremely important, may be challenging to implement. However, in order for HCL and other diabetes technologies to be rolled out on a fair and equitable basis, our findings, alongside those of others,^15^ underscore the importance of increasing staffing to prevent/alleviate staff burnout, ensure manageable workloads, and create a sustainable working environment.

Whilst advocating for increased staffing levels is crucial, consideration could also be given to the (more) cost‐effective strategies that our interviewees suggested. This includes employing non‐clinical/technical staff to free up clinical staff's time to perform more specialised tasks (e.g., insulin pump start‐ups), enlisting support from company representatives to deliver some aspects of pump training, and using group‐based formats to deliver training where possible. Across the sites, interviewees also expressed enthusiasm for employing youth and other support workers to engage and support members of underserved communities to encourage technology uptake/use. This suggestion is supported by existing literature, which has shown that individuals working with youth workers experience improved clinical outcomes and psychological benefits.^16^, ^17^

Additionally, resonating with others' findings,^18^ many interviewees highlighted benefits to conducting some ‘home visits’ and education/training sessions online as this allowed them to increase the numbers of CYP they could move onto, and support using, diabetes technology using existing (limited) capacity within their teams. As some further noted, this also permitted them to free‐up clinical capacity to support CYP who needed more intensive input to transition successfully onto technology. However, in line with some interviewees' suggestions, it is also important to recognise that, when training and support is delivered remotely, safeguards should be put in place to ensure that CYP/caregivers who experience digital poverty are not excluded from education and training opportunities. As some interviewees' accounts suggest, this might require dedicated funding to allow individuals on low‐incomes access to digital technologies (e.g., tablets or smartphones) and/or parking/travel costs to attend hospital appointments.

In many cases, interviewees appeared highly committed to addressing technology inequities and had devised solutions to help achieve this. This included working more cohesively as a team, reviewing (NPDA) data regularly, and using WhatsApp and other text‐based platforms to facilitate communication with CYP/caregivers who are not conversant in English. Free toolkits could further support teams to review data, set goals, and reduce (un)conscious bias.^19^ While these solutions have limited/no costs attached to them, other ideas interviewees proposed may be more costly or challenging to introduce and/or maintain, especially in sites with limited staffing or where staff are experiencing burnout. These included undertaking outreach work and organising peer/caregiver support (findings which align with these individuals' expressed needs^20^, ^21^) moving all CYP onto diabetes technology following diagnosis, and/or offering inpatient admissions to initiate technology use. Despite the potential challenges and costs, these solutions nevertheless merit serious consideration because, as studies have shown, supporting early diabetes technology use can promote engagement amongst those from underserved backgrounds, positively impact long‐term clinical outcomes, and reduce health disparities.^22^, ^23^, ^24^

Notably, many of the strategies interviewees reported appeared to be local (i.e., site‐specific) solutions, such as translating educational materials, developing pictorial resources, and producing video resources/testimonials, which were often made possible by short‐term, one‐off funding schemes and/or staff working additional (usually unpaid) hours. Careful consideration could therefore be given to how these existing resources might be shared/pooled to avoid unnecessary and time‐consuming duplication of work. While national/regional mechanisms for sharing these kinds of resources already exist, such as the Children's and Young People's Diabetes Network, Diabetes Technology Network UK, and NHS England Diabetes Network, most interviewees did not report using them. Hence, further consultation and collaborative work involving network leads could be beneficial. Furthermore, although such resource pooling could be advantageous, this would likely need ongoing funding (i.e., staff time) to allow resources to be maintained and/or updated and avoid the kinds of burn‐out which some interviewees indicated experiencing.

Resonating with young people's own accounts,^20^, ^25^, ^26^ interviewees emphasised the importance of offering a choice of technology to allow individual preferences to be accommodated and help address some CYP's concerns about device visibility and stigma. However, as reported previously^8^ and noted by others,^27^, ^28^ supporting a variety of different devices within a particular site can be challenging, especially when sites employ small numbers of staff and/or where staff have limited time to undertake/update device training.^5^, ^6^, ^29^, ^30^ Alongside earlier suggestions to upskill non‐clinical staff to undertake some tasks to free up clinical staff's time (e.g. to attend device training), other more ambitious solutions may also need to be considered. This might include the use of hub‐and‐spoke models and/or promoting a more mobile workforce to enable healthcare professionals with particular kinds of diabetes technology expertise (e.g., in supporting use of specific types of HCL systems) to work across different sites.

Finally, while prioritising long‐term sustainability is crucial moving forward, any future planning and strategies should be discussed with CYP/caregivers. As part of our ongoing research programme, the UNBIASED team is currently beginning work to develop recommendations and resources to promote equitable technology access using CYP/caregiver experiences and input throughout. We will report back on this work separately.

A study strength was our use of a qualitative design which enabled interviewees to offer nuanced and detailed accounts; our study was further strengthened by having three experienced qualitative researchers partake in data analysis. While we successfully recruited a diverse sample, we recognise that those who chose to take part may have been particularly motivated and/or technology‐passionate. Additionally, because our study was England‐based, some findings may not be generalisable to the rest of the UK or other countries where healthcare systems and technology access may differ.

## 5 CONCLUSION

Healthcare professionals appeared highly committed to addressing technology access disparities in CYP. While some of their recommendations may be easier to implement than others, our findings underscore the importance of adopting a joined‐up, integrated approach to promoting equitable technology access across the UK. This would require closer collaboration and resource‐sharing within and across sites, backed by sustainable, long‐term funding, with a significant portion dedicated to increasing staffing capacity to support the practical implementation of these strategies.

## AUTHOR CONTRIBUTIONS

JL conceived and designed this interview study with healthcare professionals with input from SMN, who conceived and designed the wider UNBIASED study. RD and DR collected the data, which was then analysed by RD, DR, and JL. RD and JL drafted the manuscript with input from DR. All authors reviewed, edited, and approved the final version.

## FUNDING INFORMATION

Understanding Inequalities and Barriers to Accessing Diabetes Technology in Children and Young People with Type 1 Diabetes study is funded by Diabetes UK (Grant reference number: 22/0006434) with Prof Sze May Ng, OBE as Chief Investigator/grant holder and Prof Julia Lawton, Dr. Natalie Darko, Prof Mark Evans, and Prof Nick Oliver as co‐investigators. The views expressed are those of the author(s) and not necessarily those of the funding body. The University of Cambridge has received salary support for MLE through the National Health Service in the East of England through the Clinical Academic Reserve.

## CONFLICT OF INTEREST STATEMENT

SMN declares speaker fees from Sanofi and Insulet. MLE has been a member of advisory panels and/or received speaker fees from NovoNordisk, Eli Lilly, Abbott Diabetes Care, Medtronic, Dexcom, Ypsomed, Pila Pharma, and Zucara. NO reports grants paid to their institution from the National Institute for Health and Care Research, Diabetes UK, Helmsley Trust, Dexcom, and Medtronic Diabetes; speaker fees from Tandem Diabetes, Sanofi, Dexcom, Astra Zeneca, and Medtronic Diabetes; and participation on the advisory board for Medtronic Diabetes and Roche Diabetes. RD, JL, and DR have no conflicts of interest to declare.

## CONSENT TO PARTICIPATE AND FOR PUBLICATION

All research participants provided written informed consent, including for anonymized information to be published in this article.

## Data Availability

The datasets generated and analysed for this study are not publicly available due to risks to individual privacy. However, they are available via the corresponding author on reasonable request.
